# Human-biting activities of *Anopheles* species in south-central Ethiopia

**DOI:** 10.1186/s13071-016-1813-x

**Published:** 2016-09-30

**Authors:** Oljira Kenea, Meshesha Balkew, Habte Tekie, Teshome Gebre-Michael, Wakgari Deressa, Eskindir Loha, Bernt Lindtjørn, Hans J. Overgaard

**Affiliations:** 1 Department of Zoological Sciences, Addis Ababa University, Addis Ababa, Ethiopia; 2 Department of Biology, Wollega University, Nekemte, Ethiopia; 3 Akililu Lemma Institute of Pathobiology, Addis Ababa University, Addis Ababa, Ethiopia; 4 Department of Preventive Medicine, School of Public Health, College of Health Sciences, Addis Ababa University, Addis Ababa, Ethiopia; 5 School of Public and Environmental Health, Hawassa University, Hawassa, Ethiopia; 6 Centre for International Health, University of Bergen, Bergen, Norway; 7 Norwegian University of Life Sciences, Ås, Norway; 8 Institut de Recherche pour le Développement (IRD), Maladies InfectieusesetVecteurs, Ecologie, Génétique, Evolution etContrôle (MIVEGEC), Montpellier, France; 9 Department of Entomology, Faculty of Agriculture, Kasetsart University, Bangkok, Thailand

**Keywords:** Malaria, *Anopheles arabiensis*, Endophagy, Exophagy, Ethiopia

## Abstract

**Background:**

Indoor residual spraying (IRS) and long-lasting insecticidal nets (LLINs) are the key malaria vector control interventions in Ethiopia. The success of these interventions rely on their efficacy to repel or kill indoor feeding and resting mosquitoes. This study was undertaken to monitor human-biting patterns of *Anopheles* species in south-central Ethiopia.

**Methods:**

Human-biting patterns of anophelines were monitored for 40 nights in three houses using human landing catches (HLC) both indoors and outdoors between July and November 2014, in Edo Kontola village, south-central Ethiopia. This time coincides with the major malaria transmission season in Ethiopia, which is usually between September and November. Adult mosquitoes were collected from 19:00 to 06:00 h and identified to species. Comparisons of HLC data were done using incidence rate ratio (IRR) calculated by negative binomial regression. The nocturnal biting activities of each *Anopheles* species was expressed as mean number of mosquitoes landing per person per hour. To assess malaria infections in *Anopheles* mosquitoes the presence of *Plasmodium falciparum* and *P. vivax* circumsporozoite proteins (CSP) were determined by enzyme-linked immunosorbent assay (ELISA).

**Results:**

Altogether 3,408 adult female anophelines were collected, 2,610 (76.6 %) outdoors and 798 (23.4 %) indoors. *Anopheles zeimanni* was the predominant species (66.5 %) followed by *An. arabiensis* (24.8 %), *An. pharoensis* (6.8 %) and *An. funestus* (*s.l*.) (1.8 %).

The overall mean anopheline density was 3.3 times higher outdoors than indoors (65.3 *vs* 19.9/person/night, IRR: 3.3, 95 % CI: 1.1–5.1, *P* = 0.001). The mean density of *An. zeimanni*, *An. pharoensis* and *An. funestus* (*s.l.*) collected outdoors was significantly higher than indoors for each species (*P* < 0.05). However, the mean *An. arabiensis* density outdoors was similar to that indoors (11.8 *vs* 9.4/person/night, IRR: 1.3, 95 % CI: 0.8–1.9, *P* = 0.335). The mean hourly human-biting density of *An. arabiensis* was greater outdoors than indoors and peaked between 21:00 and 22:00 h. However, *An. arabiensis* parous population showed high indoor man biting activities during bedtimes (22:00 to 05:00 h) when the local people were indoor and potentially protected by IRS and LLINs. All mosquito samples tested for CSP antigen were found negative to malaria parasites.

**Conclusions:**

Results show much greater mosquito human-biting activities occurring outdoors than indoors and during early parts of the night, implying higher outdoor malaria transmission potential in the area. However, high bedtime (22:00 to 05:00 h) indoor biting activities of parous *An. arabiensis* suggest high potential intervention impact of IRS and LLINs on indoor malaria transmission.

## Background

Malaria is the leading cause of death in wide parts of sub-Saharan Africa (SSA) [1]. Current malaria vector control in SSA relies heavily on indoor insecticidal interventions using indoor residual spraying (IRS) and long-lasting insecticidal nets (LLINs) [1–3]. The scale up of IRS and LLINs during the last decade has substantially reduced malaria incidence in many parts of SSA [4, 5]. These interventions reduce the density, feeding frequency and longevity of malaria vectors by killing the vectors with insecticides or blocking their contact with humans [6, 7] and primarily target malaria vectors that feed indoors and at night on sleeping humans [2].

However, following the adoption and scale-up of IRS and LLINs in SSA, a shift in mosquito behaviors has been observed, where mosquitoes more often bite humans outdoors and earlier in the evening, thereby avoiding insecticide treated surfaces and threatening the effectiveness of the interventions [8–11]. This behavioral change has been observed in Tanzania with *An. arabiensis* [8, 11]. On Bioko Island, Equatorial Guinea, high levels of outdoor biting by *An. gambiae* (*s.s.*) was observed throughout the night, including during early evening and morning hours when human hosts are often outdoors [9]. In Benin and Senegal, *An. funestus* has showed a behavioural change in biting activity after introduction of LLINs, remaining anthropophilic and endophilic, while adopting diurnal feeding when local people are not protected by IRS and LLINs [12, 13].

Nevertheless, in some countries in Africa, the principal vectors have shown consistent biting patterns and remain amenable to the effective IRS and LLINs interventions [14, 15]. For example, in Kenya, Bayoh et al. [15] found no evidence of behavioral shift of *An. gambiae* (*s.s.*), *An. arabiensis* and *An. funestus*from the well-known pattern of late night, indoor biting characteristics of these typically highly anthropophilic species. Based on these results, it was recommended that malaria control interventions such as LLINs should continue to be prioritized [15].

In Ethiopia, *An. arabiensis*, a member of *An. gambiae* species complex, is the sole primary vector of malaria [16]. Other *Anopheles* species such as *An. nili*, *An. funestus* and *An. pharoensis* are considered secondary vectors in the country [16]. Some evidence revealed that *An. arabiensis* and *An. funestus* were mainly endophagic and endophilic as compared to other *Anopheles* species such as *An. pharoensis*, *An. welcomi*, *An. zeimanni* and *An. nili*, which were mainly exophagic and exophilic [16–18]. *Anopheles arabiensis* were reported to bite indoors and outdoors throughout the night with peak activities at early part of the night before the inhabitants retire to bed [19–22]. This vector bites mainly on human and bovine hosts [16, 22–25]. Outdoor biting, early biting and biting on non-human hosts compromise the effectiveness of malaria vector interventions particularly IRS and LLINs that target endophagic, anthropophagic and nocturnal biting mosquitoes and worth to be monitored for evidence-based vector control interventions.

IRS has been used for more than four decades in Ethiopia [16, 26, 27]. Insecticide treated nets (ITNs) were introduced in 1997/1998 in selected malarious areas and the distribution of LLINs started in 2005 [27]. The most recent malaria strategic plan of the country is to achieve 100 % LLIN ownership per sleeping space, and 90 % IRS coverage by the end of 2015 [26]. Recently, malaria cases and deaths in Ethiopian hospitals have declined in conjunction with scale-up of malaria interventions with IRS, LLINs and artemisinin-based combination therapy (ACT) [27, 28]. Based on these successful interventions, Ethiopia is currently planning to eliminate malaria by 2020 and in light of this national target, monitoring the biting behaviour of *Anopheles* mosquitoes is crucial with respect to the efficacy of vector control interventions.

A cluster randomized trial investigating the effect of IRS and LLIN interventions combined or separate on disease outcomes has been carried out in Adami Tullu Jiddo Kombolcha district [29]. As part of this trial, the present study was undertaken to provide baseline data by monitoring local *Anopheles* species biting humans (anthropophagic anophelines) and their biting patterns in a village included in the trial.

## Methods

### The study area

**Fig. 1:**
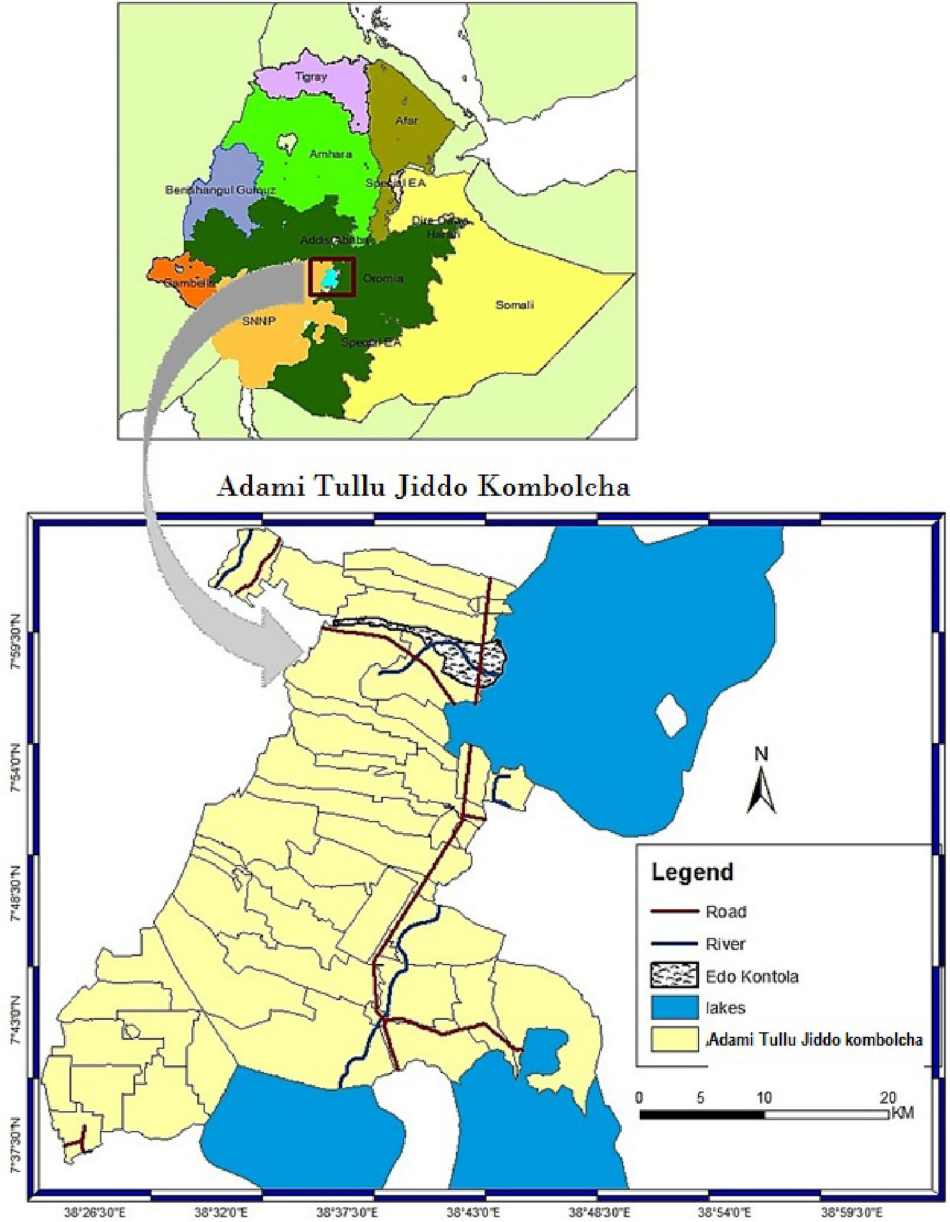
Map of Edo Kontola village, in Adami Tullu Jiddo Kombolcha district and its location in Ethiopia

This study was done in Edo Kontola village, Adami Tullu Jiddo Kombolcha district, south- central Ethiopia (Fig. 1). Edo Kontola is situated along Lake Zeway on the main road from Addis Ababa to Hawassa between Abosa and Batu towns. This village was selected based on past entomological studies [16] and recent pilot surveys [30] to study variations in biting patterns of anopheline species in the same locality and under similar environmental settings. The village is part of a cluster-randomized trial studying the effect of IRS and LLIN interventions during September 2014 and December 2016 [29]. The main environmental feature of the area is Lake Zeway which covers about 434 km^2^area with average depth of 4 m [16]. The lake supports irrigation farms and fishing, the main economic activities in the district. The people usually cultivate rain-fed maize and other cereal crops during the rainy season (June to October) and mainly vegetables such as onions, tomatoes, potatoes, and green pepper by irrigation during the dry season (November to May) and the wet season as well. Many of the inhabitants of the village live in traditional African grass-thatched house locally known as ‘mana chita’ and some live in houses with corrugated iron roofs.

The lake area maintains malaria transmission by creating and sustaining potential mosquito breeding sites specifically at the lake shoreline [31]. Mosquito abundance increases as the lake fills and extends to the nearby villages during June to October and declines as the lake volume recedes during the following dry months. The area is semi-arid causing rain-fed surface pools in the uphill villages to dry up within a short time period. As a result, mosquito breeding sites are almost limited to the lake area even during the rainy season.

Malaria transmission is seasonal and driven by seasonal precipitation. The major malaria transmission season in Ethiopia is usually September to November. The most recent study in the district indicates that of 349 blood samples taken from febrile patients, 39 (11.2 %) slides were microscopically confirmed positive for malaria infection. The overall average malaria incidence was 4.6 per 10,000 person-weeks of observation. Higher malaria incidence was observed among children < 14 years compared to older people and in villages near the lake shore than those distantly located from the lake [30]. The major malaria vector in the area is *An. arabiensis* whereas *An. pharoensis* plays a secondary role [16]. The recent pilot study results showed that *An. arabiensis* was susceptible to propoxur (carbamate), but resistant to pyrethroids. However, *An. pharoensis* was susceptible to all pyrethroids and carbamates tested [30]. The vectors transmit mainly *Plasmodium vivax* (85 %) followed by *P. falciparum* (15 %) [30]. Malaria vector control relies on LLINs and IRS. In 2012, before the trial started, 27.3 % of the households owned at least one LLINs and IRS was carried out in 91.7 % of households in the district [30]. There is one health post in the village staffed by two health extension workers. The study village has approximately 35 households with 182 people inhabiting 17,500 square meters.

### Mosquito collections

The nocturnal biting habits of *Anopheles* species were monitored for 40 nights using human landing catches (HLC) indoors and outdoors during July to November 2014, coinciding with the major malaria transmission season. The HLC, where human volunteers catch mosquitoes that land on their exposed body parts, was used because it is the gold standard method for monitoring mosquitoes that bite humans (anthropophagic mosquitoes) and the number of mosquitoes caught by HLC can directly provide an estimate of mosquito human-biting activities [32, 33].

Three houses close to the lakeshore were selected having similar size and design and with house owners agreeing to participate in the study. The houses were of traditional style with thatched conical-shaped roofs, circular floors and plastered walls. All houses had similar potential mosquito entry and exit points each having one door, eaves, and cracks in walls, but none of them had windows.

Each house in the village including the selected houses was located close to irrigation fields and within walking distance (≤ 1 km) from the lakeshore. It was also arranged in such way that the selected houses for HLC were free of cattle and human occupants on all collection nights. In addition, the houses were enrolled in the control arm of the trial and neither treated by IRS nor received LLINs during the study period [29].

The three houses were selected to reduce position bias driven by potential variations in indoor micro-climate such as indoor temperature, differences in mosquito entry points, mosquito density and proximity to animal shelter(s). Mosquito collections were performed in one house per night alternating each house for three consecutive nights per week. The collectors were rotated through the collection houses to compensate for any differences in attractiveness to mosquitoes and collecting abilities. Collections started in late July and ended in late November 2014 with intermittent collections in August and September. Mosquito collections were conducted by volunteers who were selected from the local people and who gave their written consent. Mosquitoes were collected from 19:00 to 06:00 h for 50 min each hour with 10 min rest for the volunteers. There were two collection shifts: one team of collectors worked from 19:00 to 24:00 h followed by the second team from 24:00 to 06:00 h. Every hour, two volunteers rotated between indoor and outdoor positions and carried out the work to reduce position bias. Outdoor collectors were positioned within 10 m from each study house. Each volunteer sat on a chair with the legs exposed from foot to knee and captured mosquitoes as soon as they land on the exposed legs before they commence feeding using a flashlight and mouth aspirator. Each hour’s collection was kept separately in labeled paper cups. Supervisors were assigned to coordinate collection activities and watch volunteers not to fall sleep and bitten by mosquitoes over the study nights. The next morning, mosquitoes were identified to species by morphological characteristics using the standard identification key [34], and stored on silica gel for further analysis.

Molecular identification of sibling species was not done in this study; however, *An. arabiensis* was confirmed as the only member of *An. gambiae* species complex from previous studies and from our pilot study carried out from June to October, 2013 in the area [16, 30]. From the hourly collections, fresh and unfed *An. arabiensis* (*n* = 343) were selected for ovary dissection and determination of parity based on changes in the tracheoles of the ovaries under a microscope [35].

In order to determine the *Plasmodium* infection rate, the head and thorax of each mosquito (*n* = 2,560) were carefully separated from the abdomen and tested for the presence of *P. falciparum* and *P. vivax* circumsporozoite proteins (CSP) by the direct enzyme-linked immunosorbent assay (ELISA) [36].

### Estimation of entomological parameters

Human-biting rates (HBRs) for each *Anopheles* species were calculated as mean number of mosquitoes collected by HLC per person per night (m/p/n) separately for indoor and outdoor venues, i.e. HBR = no. of mosquitoes collected/no. of nights/no. of collectors [37]. The degree of endophagy was calculated as indoor HBR_19:00→06:00 h_/(indoor HBR_19:00→06:00 h_ + outdoor HBR_19:00→06:00 h_) while exophagy was calculated as outdoor HBR_19:00→06:00 h_/(outdoor HBR_19:00→06:00 h_ + indoor HBR_19:00→06:00 h_) [38].

The density of nocturnal biting was calculated as density of HBR during peak sleeping hours (hours starting 22:00 to 05:00) as follows [38]: (indoor HBR_22:00→05:00 h_ + outdoor HBR_22:00→05:00 h_)/(indoor HBR_19:00→06:00 h_ + outdoor HBR_19:00→06:00 h_). The nocturnal biting activities of each *Anopheles* species was expressed as mean number of each *Anopheles* species landing per person per hour separated by indoor and outdoor venues. Indoor and outdoor exposure to mosquito bites that took place early evening (19:00 to 22:00 h), during night 22:00–05:00 h) and early in the morning (05:00–06:00 h) were estimated as the number of mosquito catches by HLC either indoors or outdoors divided by number of indoor and outdoor combined catches by each species multiplied by 100. Parous rate was calculated as the total number of parous females for each species divided by the total number of mosquitoes dissected multiplied by 100. The man biting proportions of parous *An. arabiensis* that took place during the early evening, during the night, and during the early morning (assessed by HLC) were compared based on field observations and available literature.

The ethical considerations for this study is described in more detail in the published protocol [29] and in the Declarations section below. In brief, to avoid the adverse effects of being bitten, mosquito collectors were trained to collect mosquitoes as soon the mosquitoes landed and before they bite. In order to minimize the risks, data collectors for the human landing catches were provided with an appropriate prophylactic drug (Malarone) before the commencement of sampling. To our knowledge, there are no reports on Malarone-resistant *Plasmodium* parasites in Ethiopia. The project provided blood examination and treatment of malaria free of charge for any study participant or householder who fell ill or wished to check himself. Fortunately, none of the mosquito collectors or householders were found parasite-positive during the study period.

### Meteorological data

Meteorological data of the study area were obtained from the Meteorological Service Agency of Ethiopia, Addis Ababa.

### Data analysis

Comparisons of indoor and outdoor HLC data were done by Generalized Linear Models (GLM) with negative binomial distribution. The impact of the collection venues on mean anopheline biting density were therefore estimated by exponentiation of negative binomial regression coefficient, i.e. Incidence Rate Ratio (IRR). Results were considered significant at *P* < 0.05. Data were analyzed using the program SPSS version 20.0 (SPSS, Chicago, USA).

## Results

### Species composition and abundance of *Anopheles* mosquitoes

During the 40 nights of human landing collections, a total of 3,408 adult female anopheline mosquitoes were captured (Table 1). *Anopheles zeimanni* was the predominant species (66.5 %), followed by *An. arabiensis* (24.8 %), *An. pharoensis* (6.8 %) and *An. funestus* (*s.l.*) (1.8 %). Overall, 76.6 % (2,610) of the mosquitoes were captured outdoors and 24.4 % (798) indoors.

**Table 1:** Total number and proportion of *Anopheles* species collected by human landing catches indoors and outdoors in Edo Kontola village, Ethiopia

Species	Indoor	Outdoor	Total
	*n* (%)	*n* (%)	*n* (%)
*An. zeimanni*	351 (15.5)	1,916 (84.5)	2,267 (66.5)
*An. arabiensis*	375 (44.4)	470 (55.6)	845 (24.8)
*An. pharoensis*	50 (21.5)	183 (78.5)	233 (6.8)
*An. funestus* (*s.l.*)	22 (34.9)	41 (65.1)	63 (1.8)
Overall	798 (23.4)	2,610 (76.6)	3,408 (100)

### Human-biting rates

The overall (indoor and outdoor combined) mean human-biting rate (HBR) of *Anopheles* mosquitoes was 85.2 mosquitoes/person/night (m/p/n). The total (indoor and outdoor combined) mean HBRs for *An. zeimanni* was 56.7, *An. arabiensis* 21.1, *An. pharoensis* 5.8, and for *An. funestus* (*s.l.*) it was 1.6 m/p/n.

**Fig. 2:**
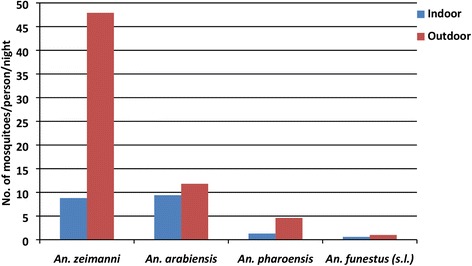
Mean indoor and outdoor human-biting rates by *Anopheles* mosquitoes in central Ethiopia

The overall mean outdoor anopheline human-biting density (HBR) was 3.3 times higher than indoor (65.3 *vs* 19.9 m/p/n, (IRR: 3.3, 95 % CI: 1.1–5.1, *P* < 0.001). The mean HBRs of *An. zeimanni*, *An. pharoensis* and *An. funestus* (*s.l.*) collected outdoors were significantly higher than indoors for each species (*P* < 0.05, Fig. 2). However, the mean outdoor HBR of *An. arabiensis* was similar to that indoors (11.8 *vs* 9.4 m/p/n, IRR: 1.3, 95 % CI: 0.8–1.9, *P* = 0.335).

**Fig. 3:**
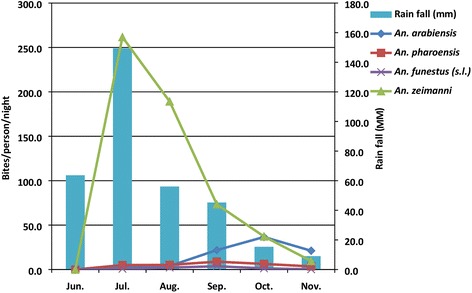
Mean human-biting rates of *Anopheles* species and average precipitation for Edo Kontola village, Ethiopia

The mean HBR of *An. zeimanni* peaked in July and declined steeply until November (Fig. 3). However, mean HBR of *An. arabiensis* peaked in October and declined thereafter. HBRs of both *An. pharoensis* and *An. funestus* (*s.l.*) were low over the study months. Monthly precipitation in the area peaked in July, and fell to its lowest in November.

### Biting behaviours: endophagy, exophagy and nocturnality

The degree of endophagy and exophagy (indoor and outdoor feeding) is given in Table 2. Overall, the majority of anophelines (76.6 %) exhibited exophagic (proportion of HBR outdoor) behaviour. The majority of *An. zeimanni* (84.5 %), *An. pharoensis* (79.3 %) and *An. funestus* (*s.l.*) (62.5 %) were captured outdoors and were clearly exophagic. For *An. arabiensis*, 55.7 % and 44.3 % of its population showed exophagic and endophagic behaviours, respectively.

**Table 2:** Human-biting rates (HBR; number of mosquitoes collected per person per night (95 % confidence interval), and feeding behaviors of *Anopheles* species in Edo Kontola village, Ethiopia

Biting activities	*An. arabiensis*	*An. pharoensis*	*An. zeimanni*	*An. funestus* (*s.l.*)	Total
Indoor HBR (19:00–06:00)	9.4 (7.9–11.0)	1.2 (0.8–1.7)	8.8 (6.1–11.6)	0.6 (0.1–1.1)	20.0
Outdoor HBR (19:00–06:00)	11.8 (9.8–14.1)	4.6 (3.6–5.6)	47.9 (38.4–56.9)	1.0 (0.6–1.5)	65.3
Nocturnal HBR (22:00–05:00)	11.2 (9.5–13.1)	2.5 (1.9–3.1)	26.5 (19.9–34.2)	0.9 (0.5–1.5)	41.1
Endophagy (%)^a^	44.3 (43.8–44.6)	20.7 (18.2–23.3)	15.5 (13.7–16.9)	37.5 (14.3–42.3)	23.4
Exophagy (%)^b^	55.7 (55.4–56.2)	79.3 (76.7–81.8)	84.5 (83.1–86.3)	62.5 (57.7–85.7)	76.6
Nocturnality (%)^c^	52.8 (44.6–53.5)	43.1 (42.4–43.7)	46.7 (44.7–49.9)	56.3 (47.6–71.4)	48.2

^a^Proportion of indoor HBR between 19:00 and 06:00 h

^b^Proportion of outdoor HBR between 19:00 and 06:00 h

^c^Proportion of HBR between 22:00 and 05:00 h (during sleeping hours)

With respect to nocturnality, overall, 48.2 % of anopheline biting occurred during peak sleeping hours (22:00 to 05:00 h) as compared to when people were most likely awake (51.8 %). None of the *Anopheles* species showed marked peak nocturnality (high nocturnal biting activities during peak sleeping hours). Similar proportion of *An. arabiensis*, *An. pharoensis* and *An. funestus* (*s.l.*) populations exhibited maximum human-biting activities during sleeping hours (50.0 %) when local people were potentially protected by LLINs and IRS as well as during non-sleeping hours (50.0 %) when the local people were not protected (Table 2).

### Human-biting patterns of anophelines and potential exposure to malaria mosquitoes

**Fig. 4:**
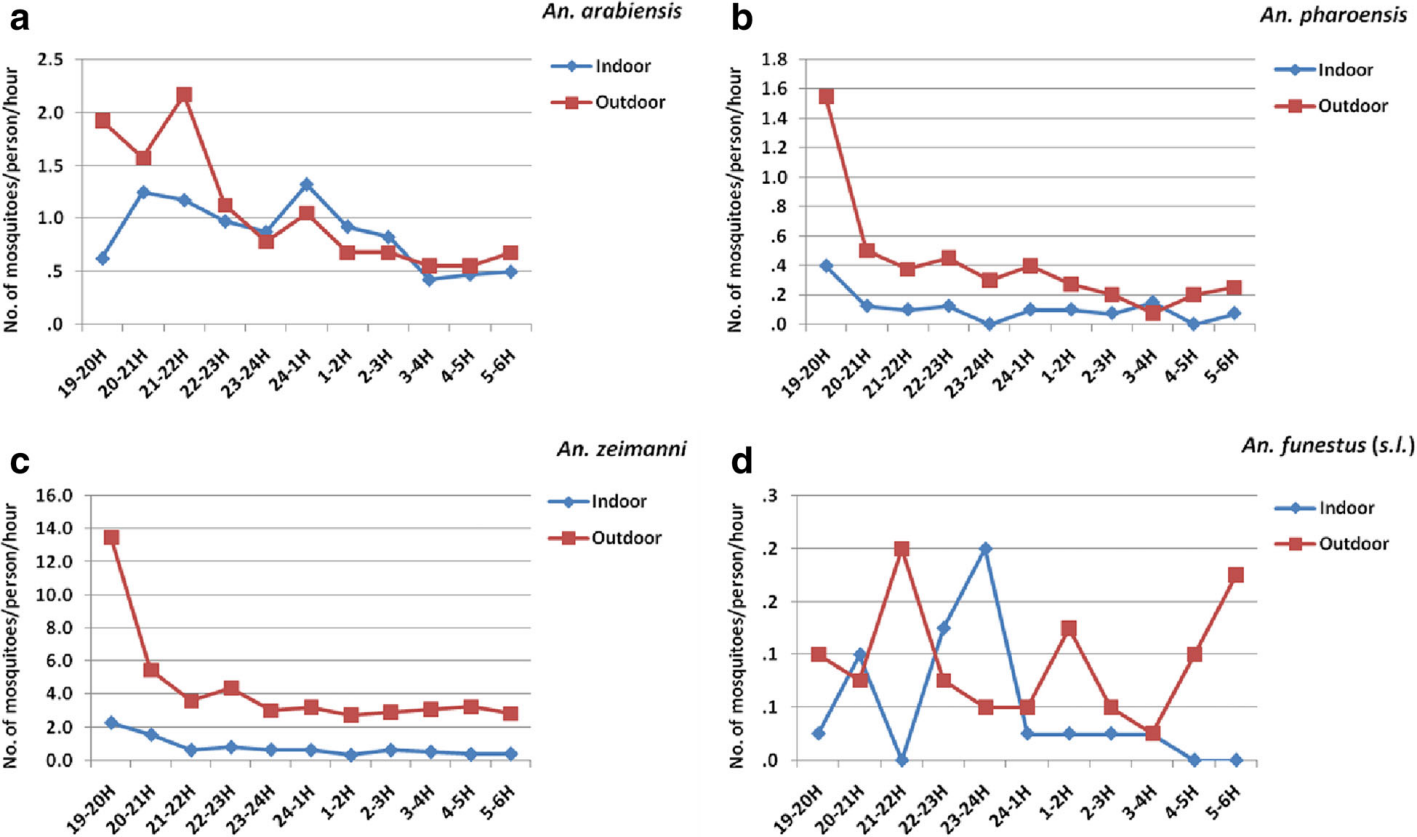
Mean hourly human-biting patterns of the *Anopheles* species in Edo Kontola, Ethiopia, 2014. **a** *An. arabiensis.* **b** *An. pharoensis.* **c** *An. zeimanni.* **d** *An. funestus* (*s.l*.)

The human-biting activity of *An. arabiensis* was from dawn to dusk both outdoors and indoors with a single peak before midnight (21:00 to 22:00 h) outdoors followed by a general decline during the rest of the night. The indoor biting activity however showed two smaller peaks, one before midnight (20:00 to 21:00 h) and a second peak around midnight (24:00 to 01:00 h) (Fig. 4a).

All the other anophelines were also active throughout the night, but with differing peak periods of biting activities both outdoors and indoors. The outdoor biting activities of both *An. pharoensis* and *An. zeimani* were generally higher than indoors and both exhibited a pronounced unimodal biting activities early in the evening (19:00 to 20:00 h) which declined progressively during the rest of the night (Fig. 4b, c), while both species also followed the same pattern indoors, but with greatly reduced biting activities. On the other hand *An. funestus* (*s.l.*) appeared to show three peaks of biting activities outdoors of which two were the major ones: one before midnight (21:00 to 22:00 h) and another one early in the morning (5:00 to 6:00 h) between which was a smaller peak just after midnight (01:00 to 2:00 h) (Fig. 4d). The indoor biting activity on the other was bimodal with an early and smaller peak at 20:00 to 21:00 h and a major peak just before midnight (23:00 to 24:00 h). Human-biting activities of the main malaria vectors; *An. arabiensis* and *An. pharoensis* peaked early in the evening (before 22:00 h) before local people retire to bed and were generally higher outdoors than indoors (Fig. 4a, b).

Altogether, 27.6 % of the major malaria vector, *An. arabiensis* bites took place during bedtime (22:00 to 05:00 h) and might be potentially prevented by LLINs alone whereas 44.4 % of this vector bites could be prevented by LLINs + IRS during the study period (Table 3). However, only 7.6 % of the potential secondary vectors, i.e. *An. pharoensis*, *An. zeimanni* and *An. funestus* (*s.l.*), bites occurred during bed time (22:00 to 05:00 h) and might be prevented by LLINs alone. Likewise, only 16.5 % of these species bites might be prevented by IRS and LLINs combined interventions.

**Table 3:** Abundance of primary (*An. arabiensis*) and secondary [*An. pharoensis*, *An. zeimanni*, *An. funestus* (*s.l.*)] malaria vectors collected indoors and outdoors at different times of the night in Edo Kontola village, Ethiopia

*Anopheles* species	Venue	Early evening (19:00–22:00 h) *n* (%)	Night (22:00–05:00 h) *n* (%)	Early morning (05:00–06:00 h) *n* (%)	Whole night (19:00–06:00 h) *n* (%)
Primary vector	Indoor	122 (14.4)	233 (27.6)	20 (2.4)	375 (44.4)
	Outdoor	227 (26.8)	216 (25.6)	27 (3.2)	470 (55.6)
Secondary vectors	Indoor	208 (8.1)	196 (7.6)	19 (0.7)	423 (16.5)
	Outdoor	1,014 (39.6)	996 (38.8)	130 (5.1)	2,140 (83.5)

Percentages were calculated as the number of mosquito catches by HLC, either indoor or outdoor, divided by the number of indoor and outdoor combined catches

### Human-biting patterns of parous *An. arabiensis* population

In total 343 *An. arabiensis* were dissected to determine parity rates and man biting patterns of the parous population. The overall indoor parous rate of *An. arabiensis* was 70.6 % and the corresponding outdoor parous rate was 67.5 % (Table 4). The proportion of parous *An. arabiensis* population that showed indoor man biting activities during bedtimes (22:00 to 05:00 h) when the local people were indoors and potentially protected by IRS and LLINs was 72.4 %. Likewise 69.2 % of parous *An. arabiensis* were collected while attempting to bite man before bedtimes (before 22:00 h). The proportion of parous *An. arabiensis* (50.0 %) caught biting people during early morning (05:00 to 6:00 h) was low compared to either before bedtime or during bedtime. The overall indoor parous rate of *An. arabiensis* was high (76.1 %) in October and low (54.5 %) in November. The corresponding outdoor parous rate was high (75.0 %) in September and low (45.7 %) in November. No ovarial dissection was carried out in July and August due to low mosquito density.

**Table 4:** Parity rates (in %) of *An. arabiensis* (no. of parous/no. of tested) collected by human landing collections indoors and outdoors at different times of the night during three months in Edo Kontola village, Ethiopia

Month	Venue	Time			Total
		Early evening (19:00–22:00)	Night (22:00–05:00)	Early morning (05:00–06:00)	Whole night (19:00–06:00)
September	Indoor	58.3 (7/12)	69.2 (9/13)	0 (0/0)	72.7 (16/22)
	Outdoor	40.0 (2/5)	84.6 (11/13)	100.0 (2/2)	75.0 (15/20)
October	Indoor	77.8 (28/36)	76.0 (38/50)	50.0 (1/2)	76.1 (67/88)
	Outdoor	71.8 (56/78)	73.3 (44/60)	57.1 (4/7)	71.7 (104/145)
November	Indoor	50.0 (10/20)	61.5 (8/13)	0 (0/0)	54.5 (18/33)
	Outdoor	62.5 (10/16)	33.3 (6/18)	0 (0/1)	45.7 (16/35)
Total	Indoor	69.2 (45/65)	72.4 (55/76)	50.0 (1/2)	70.6 (101/143)
	Outdoor	68.7 (68/99)	67.0 (61/91)	60.0 (6/10)	67.5 (135/200)

### Malaria infection

A total of 1,500 *An. zeimanni*, 800 *An. arabiensis*, 200 *An. pharoensis* and 60 *An. funestus* (*s.l.*) were tested for the presence of CSP of *P. falciparum* and *P. vivax*. However, none was found positive. For this reason, the entomological inoculation rate (EIR) could not be determined.

## Discussion

*Anopheles zeimanni*, *An. arabiensis*, *An. pharoensis* and *An. funestus* (*s.l.*) were found to be the four human-biting anopheline species occurring indoors and outdoors in the study area. This finding is similar to a recent pilot study [30] and previous entomological collections in the same area [16]. These results also showed that *An. zeimanni* (member of the *An. coustani* species complex) was the most predominant species and outnumbered the primary malaria vector, *An. arabiensis* which was once the most abundant species [16]. Furthermore, *An. arabiensis* was the only member of *An. gambiae* complex [16, 30]. More recently, Bekele et al. [39] reported that *An. zeimanni* was among the most abundant anopheline species in three villages near the study village. The abundance of *An. zeimanni* observed in the present study agrees with the earliest reports by Krafsur [17] who recorded that this species was the predominant species biting man near swamp margins in Gambela Region, western Ethiopia.

The abundance of *An. zeimanni* over *An. arabiensis* could be attributed to difference in their breeding site preferences. The present study village, Edo Kontola is located along Lake Zeway on the margin of swamp associated with aquatic vegetation that might have favored abundance of *An. zeimanni* that typically breed in vegetative swamps. *Anopheles zeimanni* has been found to be closely associated with aquatic vegetation [17] whereas *An. arabiensis* typically breeds in small, sunlit temporary water pools [31]. Another potential reason could be inter species differences in feeding behaviours between *An. zeimanni* and *An. arabiensis*. Results showed that man-biting densities of *An. zeimanni* were significantly higher outdoor than indoor and exhibited more exophagic than endophagic behaviour unlike *An. arabiensis* which was a more endophagic species. This finding is consistent with previous reports in the country [16, 17]. Because *An. zeimanni* was more exophagic than *An. arabiensis*, it appears to have less chance to come in contact with LLINs and IRS. This might have favored *An. zeimanni*, while *An. arabiensis* population was affected by IRS and LLINs interventions. Although *An. zeimanni* has not been incriminated as a malaria vector in Ethiopia so far, it is a locally important vector in Cameroon [40]. In Ethiopia, a single malaria-infected *An. zeimanni* was observed in the western part of the country [41]. Its higher abundance and human-biting activities observed in the current study also imply that it has a potential role in malaria transmission. More studies are required on the infectivity, vectorial capacity and competence, ecology, and bionomics of *An. zeimanni*.

The present results also revealed that the *Anopheles* mosquitoes, in general, bite more frequently outdoors than indoors. Because, both IRS and LLINs are indoor-based, high outdoor human-biting rates indicate possible outdoor malaria transmission potential in the area. These findings might compromise the efficacy and effectiveness of LLINs and IRS and point to the necessity of outdoor vector interventions. The results show evidence for the occurrence of residual malaria transmission potential but the magnitude and impact of such transmission warrants further investigation in the area and elsewhere in the country. *Anopheles pharoensis* exhibited more exophagic behaviour than endophagic behaviour. These results would be expected because, *An. pharoensis* is an exophagic species in Ethiopia [16–18] and elsewhere in Africa [42].

Unlike the other anopheline species, there were no significant differences in outdoor and indoor human-biting rates of *An. arabiensis*. This indicates a high flexibility and plasticity of the vector with respect to indoor and outdoor feeding and potential host preferences. Previous studies show that *An. arabiensis* bite both indoors and outdoors [16–18]. With respect to host preference, *An. arabiensis* has shown opportunistic feeding behaviour in Ethiopia [25], exhibiting either anthropophagic [23, 30, 43] or zoophagic behaviour [24]. This study did not look for host preferences because mosquito collections were done by HLC alone, which is an unsuitable method for blood meal source analysis.

Analysis of the biting patterns showed early-evening biting behaviour of *An. arabiensis* with the highest peak occurring before 22:00 h indoors and outdoors at times when the local people are not protected by LLINs. We have observed that villagers, both children and adults, spend time outdoors performing various activities such as fishing, looking after their cattle and typically retire to bed after 22:00 h. Previous reports also indicated that the people retire to bed after 22:00 h [16]. These human activities can increase exposure to mosquito bites. Previous studies in the same study area [16, 20] and elsewhere in the country [21, 22] have also recorded early biting behaviour of *An. arabiensis*. In contrast to the present results, some findings documented peak *An. arabiensis* man-biting activities after 23:00 h [18]. In short, the previous and the present results suggest that *An. arabiensis*'s behaviour is flexible and potentially opportunistic in terms of host preference, and feeding and resting habits [16, 18–25].

These flexible behaviours remain a key challenge for malaria control and elimination because the vector may be less vulnerable to IRS and LLINs, and as a result, may sustain malaria transmission. Although these behaviours are believed to be a consequence of long-term exposure to IRS and LLINs interventions in Ethiopia [21, 23], evidence is still lacking. Sufficient historical and up-to-date evidence about the impact of insecticidal interventions on *An. arabiensis* population and behaviour is needed to suggest that the vector is showing behavioural adaptation or has consistent biting patterns in the country. These issues need special attention for malaria control and elimination efforts in the country.

The peak indoor and outdoor man-biting activities of *An. pharoensis* and *An. zeimanni* occurred during early hours of the evening and there has been no evidence of behavioural modifications or shifts. These results are in agreement with other studies undertaken in this area [16]. *Anopheles funestus* (*s.l.*) did not show clear indoor and outdoor human-biting patterns due to small numbers collected.

The overall indoor parity rate for *An. arabiensis* was 70.6 % and is similar to earlier reports from the same area by Rishikesh [44] who recorded a constant parity rate ranging from 65–70 % for *An. gambiae* (*s.l.*) presumably *An. arabiensis*. With this parity rate, *An. arabiensis* lived long enough to maintain indoor malaria transmission. Results show that indoor parity rates of *An. arabiensis* were high at times when local people generally are asleep indoors and potentially under LLINs [16]. This implies that IRS and LLINs have high potential intervention impact on indoor malaria transmission.

Results also show that all mosquito samples tested by ELISA (*n* = 2,560) were negative for *P. falciparum* and *P. vivax* circumsporozoite protein infection. It is not uncommon to find sporozoite negative mosquito samples in areas with seasonal malaria transmission such as in this study area [16]. Low sporozoite infection rates have been repeatedly reported from the study area, for example, Rishikesh [44] found nine sporozoite-positive mosquitoes (0.2 %) out of 4,513 *An. gambiae* (*s.l.*) (*An. arabiensis*) dissected for salivary gland examination. Kibretet al. [20] also found 0.6 % and 1.2 % *P. falciparum* sporozoite rates among 509 *An. pharoensis* and 424 *An. arabiensis*, respectively, collected by CDC light traps and tested by ELISA in an irrigated village in the proximity of Zeway Lake. In contrast, no sporozoite-positive mosquitoes were detected in a non-irrigated village located relatively far from the lake [20]. The current malaria decline coinciding with the scale-up of vector interventions and malaria treatment measures in the country [28, 45] might have reduced malaria parasites in the mosquito population. Furthermore, it can be suggested that lack of large numbers of mosquito specimens due to low mosquito density in the area and lack of access to more sensitive sporozoite testing methods than ELISA (such as quantitative real-time PCR) to detect infective mosquitoes could be potential factors for the negative results.

## Conclusions

*Anopheles zeimanni*, *An. arabiensis*, *An. pharoensis* and *An. funestus* (*s.l.*) were found to be the human-biting species in the area, all with outdoor biting behaviours. A high proportion of parous *An. arabiensis* were collected during night times, when the local people are usually indoors and potentially protected by IRS and LLINs. These results suggest that: (i) early and outdoor biting behaviour of *An. arabiensis* could compromise the effectiveness of IRS and LLINs and point to the need for complementary interventions, and (ii) IRS and LLINs still have an impact on indoor malaria transmission suggesting that application and adherence to these interventions need to be strengthened.

## Abbreviations

HLC: Human landing catches of mosquitoes arriving to feed at humans
IRS: Indoor residual spraying of household walls with insecticides
LLINs: Long-lasting insecticide treated nets.
